# Human Cytomegalovirus (HCMV)-Specific CD4^+^ and CD8^+^ T Cells Are Both Required for Prevention of HCMV Disease in Seropositive Solid-Organ Transplant Recipients

**DOI:** 10.1371/journal.pone.0106044

**Published:** 2014-08-28

**Authors:** Elisa Gabanti, Francesca Bruno, Daniele Lilleri, Chiara Fornara, Paola Zelini, Ilaria Cane, Clara Migotto, Eleonora Sarchi, Milena Furione, Giuseppe Gerna

**Affiliations:** 1 Laboratori Sperimentali di Ricerca, Area Trapiantologica, Fondazione IRCCS Policlinico San Matteo, Pavia, Italy; 2 Divisione di Nefrologia, Fondazione IRCCS Policlinico San Matteo, Pavia, Italy; 3 Divisione di Cardiochirurgia, Fondazione IRCCS Policlinico San Matteo, Pavia, Italy; 4 S. S. Virologia Molecolare, S. C. Microbiologia e Virologia, Fondazione IRCCS Policlinico San Matteo, Pavia, Italy; University of San Francisco, United States of America

## Abstract

In solid-organ transplant recipients (SOTR) the protective role of human cytomegalovirus (HCMV)-specific CD4^+^, CD8^+^ and γδ T-cells vs. HCMV reactivation requires better definition. The aim of this study was to investigate the relevant role of HCMV-specific CD4^+^, CD8^+^ and γδ T-cells in different clinical presentations during the post-transplant period. Thirty-nine SOTR underwent virologic and immunologic follow-up for about 1 year after transplantation. Viral load was determined by real-time PCR, while immunologic monitoring was performed by measuring HCMV-specific CD4^+^ and CD8^+^ T cells (following stimulation with autologous HCMV-infected dendritic cells) and γδ T-cells by flow cytometry. Seven patients had no infection and 14 had a controlled infection, while both groups maintained CD4^+^ T-cell numbers above the established cut-off (0.4 cell/µL blood). Of the remaining patients, 9 controlled the infection temporarily in the presence of HCMV-specific CD8^+^ only, until CD4^+^ T-cell appearance; while 9 had to be treated preemptively due to a viral load greater than the established cut-off (3×10^5^ DNA copies/mL blood) in the absence of specific CD4^+^ T-cells. Polyfunctional CD8^+^ T-cells as well as Vδ2^−^ γδ T-cells were not associated with control of infection. In conclusion, in the absence of HCMV-specific CD4^+^ T-cells, no long-term protection is conferred to SOTR by either HCMV-specific CD8^+^ T-cells alone or Vδ2^−^ γδ T-cell expansion.

## Introduction

The immune response to human cytomegalovirus (HCMV) infection involves both humoral and T-cell responses in primary as well as reactivated (recurrent) infections. The antibody (both neutralizing and ELISA) response occurs early reaching high levels in primary as well as in recurrent infections [1]–[3]. However, the major role of T-cell-mediated immunity against recurrent infections has been documented in solid-organ transplant recipients (SOTR), in whom the absence of T-cell immunity reconstitution after transplantation is associated with high viral load levels in peripheral blood and a high frequency of HCMV disease, often in the presence of high neutralizing antibody levels.

Although the pivotal role of T-cell immunity in protection against HCMV disease in the post-transplant period is well established, the relative impact of HCMV-specific CD4^+^ and CD8^+^ T-cells remains to be defined. Initially, it was believed that the cytotoxic/cytolytic activity of specific CD8^+^ T-cells was predominant in protection against HCMV recurrence both in mice and man [4]–[6]. Subsequently, the helper role of HCMV-specific CD4^+^ T-cells was reevaluated utilizing the murine CMV model of infection [7] as well as in man, both in the immunocompetent and immunocompromised host [8]–[11]. Moreover, γδ T-cells (in particular the Vδ2^−^ subset) were reported to be implicated in the control of HCMV infection [12]–[14]. However, at this time, the relative role of HCMV-specific CD4^+^, CD8^+^ and γδ T-cells in protection against HCMV replication relapse has not been clearly defined at the clinical level.

The main objective of this study was to retrospectively define the role of HCMV-specific CD4^+^ T-cells in combination with HCMV-specific CD8^+^ T-cells and γδ T-cells in the control of HCMV infection reactivation in a series of 39 HCMV-seropositive SOTR displaying different clinical presentations with respect to HCMV infection, i.e. i) lack of infection, ii) stable control of infection (in the presence of stable levels of HCMV-specific CD4^+^ and CD8^+^ T-cells), iii) transitory control of infection in the presence of HCMV-specific CD8^+^ only, until CD4^+^ T-cell appearance, and iv) lack of control with high viral load requiring antiviral treatment in the presence of HCMV-specific CD8^+^, but in the absence of CD4^+^ T-cells.

## Patients and Methods

### Study population

From June 2011 to July 2012, 64 HCMV-seropositive patients receiving a kidney (n = 40) or heart (n = 24) transplantation at the University Hospital, Fondazione IRCCS Policlinico San Matteo, Pavia, Italy, were enrolled in the study. Among SOTR, 25 patients were excluded from the analysis because of: i) early death (within 1 month after transplantation) for causes not related to HCMV infection (n = 9); ii) post-surgical follow-up performed in other centers (n = 15); and iii) non-compliance with virological follow-up (n = 1). Thus, 25 kidney (KTR) and 14 heart (HTR) transplant recipients were analysed. Median age was 55 (range 42–71) years for KTR, and 54 (range 24–65) years for HTR. Median follow-up time was 365 days (range 192–405) for HTR, and 356 days (range 114–497) for KTR. HTR received induction therapy with anti-thymocyte globulin (ATG) and steroids, whereas KTR received either ATG and steroids or anti-CD25 monoclonal antibody and steroids. Immunosuppressive therapy consisted of standard triple therapy including a calcineurin inhibitor (cyclosporine A or tacrolimus), an antiproliferative drug (mophetil mycophenolate, MMF) or an mTOR inhibitor (everolimus), and steroids. In 6/25 (24.0%) KTR, everolimus (the rapamycin derivative RAD) was added to the standard triple therapy (with a low MMF dosage), while in 5/14 (35.7%) HTR RAD was administered instead of MMF. Patients with organ rejection episodes were treated with a daily bolus of intravenous methylprednisolone (1 g or 500 mg) for three days. Thirty HCMV-seropositive adult healthy subjects were used as controls. The study was approved by the Fondazione IRCCS Policlinico San Matteo Institutional Review Board (IRB, Protocol n. 20100005459, Procedure n. 20100035853), and patients gave written informed consent prior to entering the study.

### Monitoring of HCMV infection

HCMV infection (virus detection in blood or tissues) and disease (HCMV infection in association with clinical symptoms and/or organ dysfunction) were diagnosed by real-time PCR, as reported [15], [16]. Viral DNA was quantified in blood weekly or bi-weekly concomitantly with active HCMV infection. Pre-emptive therapy was given to patients reaching a peripheral blood cut-off of 300,000 DNA copies/mL as previously reported [17]–[19]. In some patients, clinicians started therapy after reaching 100,000 DNA copies/mL blood due to detection of virus within organs [20]. Antiviral therapy consisted of intravenous ganciclovir administration at a dosage of 5 mg/kg twice a day or valganciclovir (VGCV), 900 mg twice a day. Antiviral treatment was continued until virus disappearance from blood. If required, HCMV infection relapses were similarly treated.

### Immunologic monitoring

Immunologic monitoring was performed at day 0 (immediately before transplantation), and 30, 60, 90, 120, 180, and 360 days after transplantion, unless otherwise indicated by the clinical condition. HCMV-specific CD4^+^ and CD8^+^ T-cells were stimulated with autologous monocyte-derived HCMV VR1814-infected dendritic cells (iDC), as reported [21]. Then, iDC were resuspended in 100 µL RPMI 1640 and incubated overnight at 37°C in a 5% CO_2_ atmosphere with 0.5–1.0×10^6^ PBMC aliquots in the presence of brefeldin A (10 µg/mL). In parallel, PBMC were stimulated with mock-infected autologous immature DC, following the same procedure. Based on testing of a series of HCMV-seropositive and HCMV-seronegative healthy blood donors, subjects with virus-specific cellular immunity were those with more than 0.4 HCMV-specific CD4^+^ and CD8^+^ T-cells/µL blood [21]. The same cut-offs were found to be valid also for SOTR [22]. Similar cut-offs have been reported by others [23].

### Flow cytometry analysis

Absolute CD3^+^CD4^+^ and CD3^+^CD8^+^ T-cell counts were measured in whole blood samples by direct immunofluorescence flow cytometry (TruCOUNT tubes, BD Biosciences, San Jose, CA, USA). In addition, γδ T-cells were measured in whole blood samples incubated with a mix of the following mAbs: APC-Cy7-conjugated anti-CD45 (clone 2D1), Pacific Blue-conjugated anti-CD3 (clone UCHT1) (BD Biosciences, San Jose, CA, USA), FITC-conjugated anti-TCR Vδ2 (clone IMMU 389), PE-conjugated anti-TCR pan γδ (clone IMMU 510) (Beckman Coulter Immunotech, Marseille, France). The total number of Vδ2^+^ and Vδ2^−^ T-cells was determined by multiplying the percentages of γδ T-cell subsets by the absolute lymphocyte count determined in whole blood.

For determination of HCMV-specific T-cells, following incubation with iDC, cells were washed and then incubated with Live/Dead Fixable Violet Dye (Invitrogen) and V500-conjugated anti-CD8 (clone RPA-T8) for cell surface staining. Cells were then washed and permeabilized (FACS Permeabilizing Solution, BD Biosciences) and incubated with an intracellular mix of the following mAbs: PerCP-Cy5.5-conjugated anti-CD3 (clone UCHT1), APC-Cy 7 -conjugated anti-CD4 (clone RPA-T4), PECy 7-conjugated anti-IFN-γ (clone B27), APC-conjugated anti-IL-2 (clone MQ1–17H12), FITC-conjugated anti-TNF-α (clone 6401.1111) (BD Biosciences). Finally, cells were washed, resuspended in 1% paraformaldehyde and analyzed with a FACSCanto II flow cytometer (BD Biosciences). As a routine, 1–2×10^5^ viable lymphocytes were collected and at least 2.5×10^4^ CD3^+^ CD4^+^ and CD3^+^ CD8^+^ T-cells were analyzed. The frequency of CD4^+^ and CD8^+^ T-cells producing IFN-γ, TNF-α and IL-2 in response to the iDC stimulus was determined by subtracting the frequency of control cells incubated with mock-infected DC (<0.05%) from the test frequency. The total number of HCMV-specific CD4^+^ and CD8^+^ T cells was calculated by multiplying the percentages of HCMV-specific T-cells by the relevant absolute CD4^+^ and CD8^+^ T-cell counts.

### Statistical analysis

The number of HCMV-specific CD4^+^ and CD8^+^ T-cell responses to iDC, as well as the number of T-cells producing different cytokines were compared by using the Mann-Whitney *U* test. The Kruskall-Wallis test was used to compare more than two groups, with Dunn’s post-test and correction for multiple comparisons. Receiver-Operator Characteristic (ROC) analysis was used to study the correlation of absolute and HCMV-specific CD4^+^ and CD8^+^ T-cells and control of HCMV infection. The association of different factors with the patient distribution into four groups was analyzed by the Chi-square test. The Wilcoxon test for paired data was used to compare polyfunctional and monofunctional subsets in healthy controls and in transplanted patients.

## Results

### Different patterns of HCMV infection in SOTR

**Figure 1:**
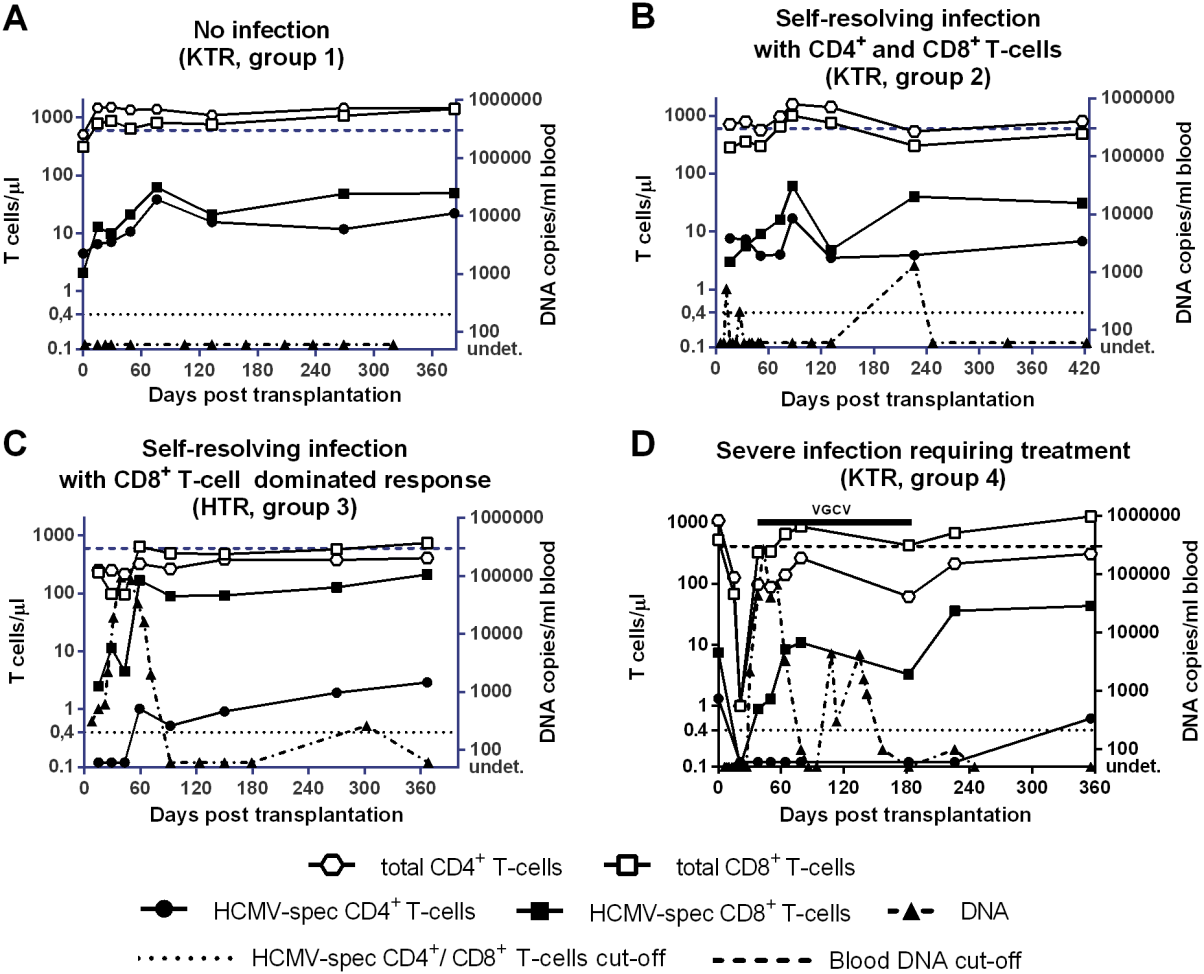
Kinetics of absolute numbers/µl blood of total and HCMV-specific CD4^+^ and CD8^+^ T-cells in four SOTR patients (each representative of one of the four patient groups). Patient A (group 1): no HCMV infection (no viral DNA) is detected and HCMV-specific CD4^+^ and CD8^+^ T-cells are consistently above the cut-off (black dotted line corresponding to 0.4 T-cells/µl blood); Patient B (group 2): self-resolving infection in the presence of low viral load and specific CD4^+^ and CD8^+^ T-cells consistently above the cut-off; Patient C (group 3): self-resolving infection in the presence of a high viral load peak and a number of HCMV-specific CD8^+^ T-cells above the cut-off, but in the absence of specific CD4^+^ T-cells or in the presence of CD4^+^ T-cells at a level close to the cut-off for the first two-three months after transplantation; Patient D (group 4): uncontrolled infection in the presence of high viral load above the cut-off (requiring antiviral treatment) and absence of specific CD4^+^ T-cells until 12 months after transplantation. The dashed line indicates the cut-off of viral load to start preemptive therapy. KTR, kidney transplant recipient; HTR, heart transplant recipient; VGCV, valganciclovir.

On the basis of clinical and virologic (viral load) monitoring, the 39 SOTR analyzed in this study were divided into four groups (Table 1). The kinetics of viral load as well as the total and HCMV-specific CD4^+^ and CD8^+^ T-cell responses for a single patient representative of each of the four groups are illustrated in Fig. 1A–D.

**Table 1:** Virological and immunological monitoring of the four groups of solid-organ transplant recipients with or without HCMV infection reactivation.

Follow-up(days)	T-cell count/µl blood median (range)								HCMV infection			
	Absolute				Specific				Onset	Duration	DNA peak	
	CD4^+^		CD8^+^		CD4^+^		CD8^+^		Median daysPosttx (range)	Median days (range)	Mediancopies/mlblood (range)	Median days post tx (range)
	Onset^1^	End^1^	Onset	End	Onset	End	Onset	End				
**Group 1 (no HCMV reactivation, n = 7, 6 KTR and 1 HTR)**												
1–365	604	542	548	703	4.8	2.9	2.2	40.0	NA	NA	NA	NA
	(257–1,515)	(191–1,458)	(239–879)	(267–1,414)	(0.5–10.5)	(0.7–63.8)	(0.5–94.8)	(3.2–87.3)				
**Group 2 (HCMV reactivation controlled by specific CD4^+^ and CD8^+^ T-cells, n = 14, 12 KTR and 2 HTR)**												
1–365	488	677	300	573	3.6	5.9	8.7	67.7	34	120	1,300	58
	(832,159)	(228–911)	(58–1154)	(253–1,862)	(0.1–16.4)	(0.4–23.8)	(0.1–87.1)	(18.3–220.8)	(12–113)	(6–363)	(200–35,000)	(14–226)
**Group 3 (HCMV reactivation temporarily controlled by specific CD8^+^ T-cells, n = 9, 4 KTR and 5 HTR)**												
1–90^2^	506	535	218	452	0.1	0.6	0.4	22.3	30	116	10,200	44
	(107–1,003)	(167–1,652)	(92–372)	(133–1,363)	(0.1–0.3)	(0.1–6.8)	(0.1–11.4)	(0.5–90.5)	(6–44)	(30–399)	(1,950–259,000)	(18–52)
91–365^2^	444	394	554	597	0.8	0.7	27	55.9			350	110
	(232–1,685)	(189–1,446)	(329–1,136)	(159–870)	(0.4–1.3)	(0.3–2.9)	(8.4–66.3)	(21–212.6)			(<25–287,200)	(91–346)
**Group 4 (uncontrolled HCMV reactivation and need for antiviral treatment, n = 9, 3 KTR and 6 HTR)**												
1–365	198	401	195	1021	0.1	0.7	0.8	29.5	25	182	308,000^3^	48
	(22–975)	(250–1,118)	(60–815)	(727–2,391)	(0.1–0.9)	(0.1–6.7)	(0.1–8.7)	(5.3–323.5)	(14–37)	(50–347)	(119,900–858,000)	(41–136)

NA, not applicable.

1 Follow-up.

2 Patients of this group showed HCMV specific CD8^+^ only, until about 3 months after transplantation, then developed HCMV-specific CD4^+^ T-cells.

3 In some cases, clinicians preferred initiating antiviral therapy after reaching 100,000 (instead of 300,000) DNA copies/ml blood due to presence of end-organ disease.

Group 1 included 7 patients (6 KTR and 1 HTR, 4 D^+^R^+^, and 3 R^+^ with D unknown) with a median age of 50 (range 43–60) years, who did not present with HCMV infection in the post-transplant period. Viral load was negative (below the PCR limit of detection of 25 copies/ml blood). These patients maintained median levels of total CD4^+^ and CD8^+^ T-cells above 500 cells/µL and levels of HCMV-specific CD4^+^ and CD8^+^ T-cells above the in-house established cutoffs of 0.4 T-cells/µL during the entire follow-up (see representative case in Fig. 1A). At the onset of follow-up, i.e. 30 days after transplantation, medians of specific CD4^+^ T-cells were 4.8 (0.5–10.5) and CD8^+^ T-cells 2.2 (0.5–94.8), while, at the end of follow-up, medians of CD4^+^ T-cells were 2.9 (0.7–63.8) and CD8^+^ T-cells were 40.0 (3.2–87.3). However, the CD8^+^ T cell increase was not significant throughout the study period.

**Figure 2:**
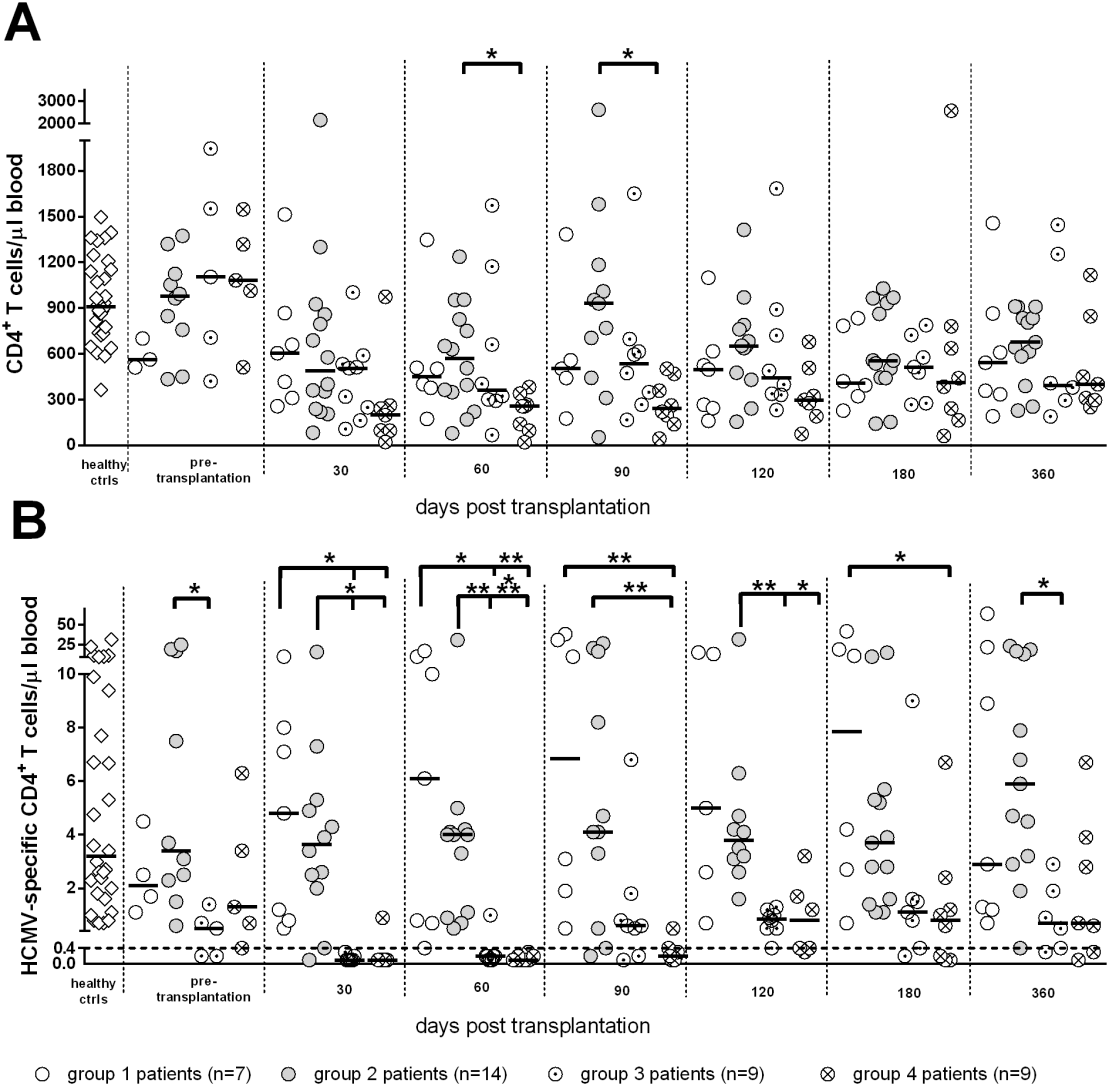
Kinetics of median levels of (A) total and (B) HCMV-specific CD4^+^ T-cells/µl blood in SOTR. A significantly higher number of HCMV-specific CD4^+^ T-cells was found in groups 1 and 2 *vs* groups 3 and 4 at 30 and 60 days after transplantation, and in groups 1 and 2 *vs* group 4 at 90 days after transplantation. *, P<0.05; **, P<0.01; ***, P<0.001.

**Figure 3:**
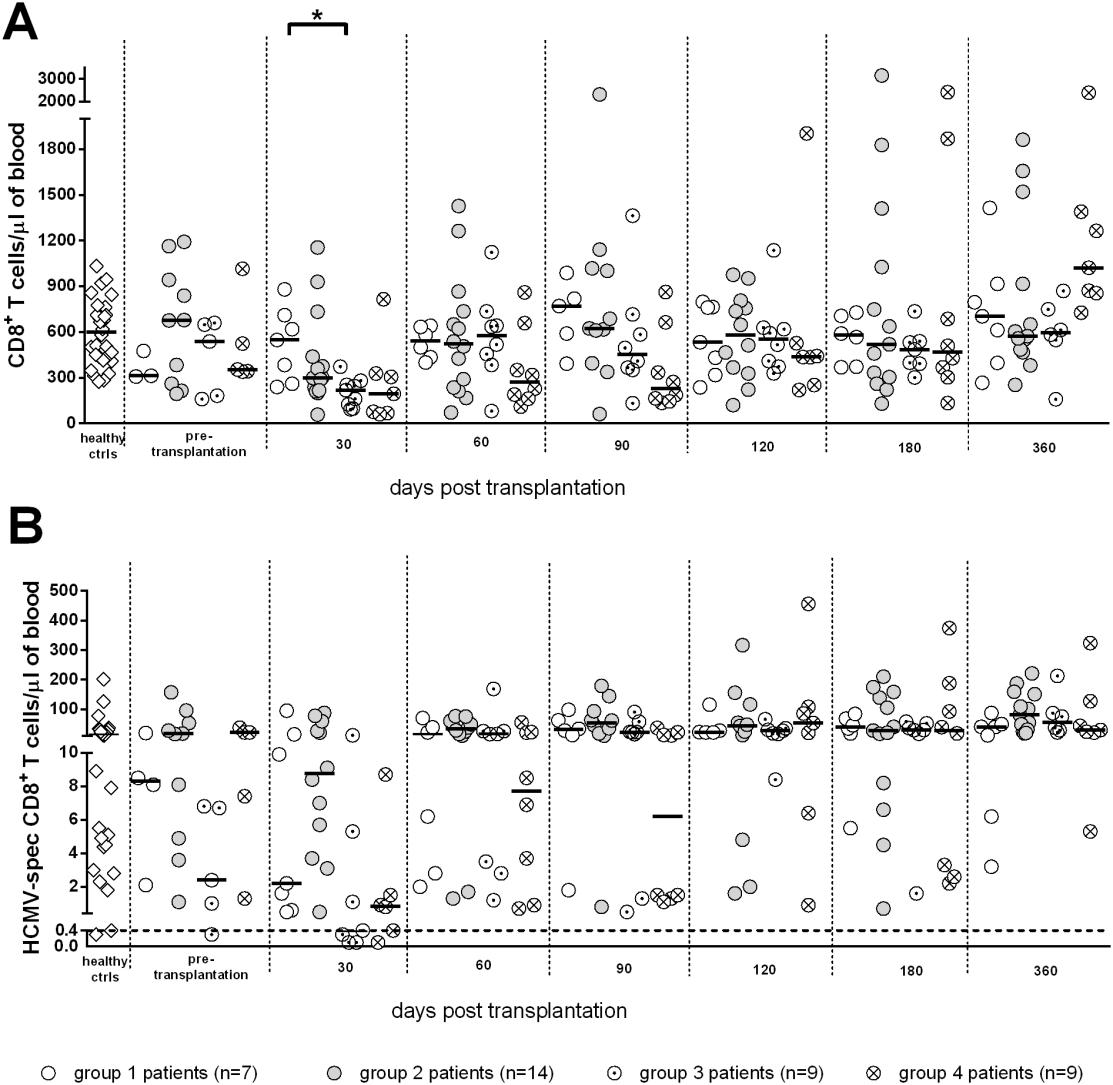
Kinetics of median levels of (A) total and (B) HCMV-specific CD8^+^ T-cells in SOTR. No significant difference was found in the number/µl blood of HCMV-specific CD8^+^ T-cells among the different groups at any time post-transplant.

Group 2 included 14 patients (12 KTR and 2 HTR, 9 D+R^+^, 1 D^−^R^+^, and 4 R^+^ with D unknown) with a median age of 57 (46–71) years, who underwent self-resolving HCMV infection during the post-transplant period, never reaching the viral load cutoff established for initiation of pre-emptive therapy [median HCMV DNA peak level was 1,300 (200–35,000) copies/mL blood]. Viral DNA appeared in blood at a median time of 34 (12–113) days after transplantation, and was detected for a median duration of 120 (6–363) days, reaching its peak 58 (14–226) days after transplantation. Group 2 patients consistently controlled viral infection in the presence of median levels of total CD4^+^ and CD8^+^ T-cells above 300/µL and levels of specific T-cells above the cutoffs of 0.4 cells/µL blood (see representative case in Fig. 1B). These levels were not significantly different from those of group 1 patients (Fig. 2 and 3). HCMV-specific CD4^+^ and CD8^+^ T-cells were 3.6 (0.1–16.4) and 8.7 (0.1–87.1) at the onset, and 5.9 (0.4–23.8) and 67.7 (18.3–220.8)/µL blood at the end of follow-up, respectively. However, both CD4^+^ and CD8^+^ T cell increases were not significant throughout the study period.

Group 3 included 9 patients (4 KTR and 5 HTR, 6 D^+^R^+^, and 3R^+^ with D unknown) with a median age of 58 (range 24–69) years, who showed a median peak HCMV DNA level of 10,200 (range 1,950–259,000) copies/mL blood, far below the cutoff for preemptive therapy. In these patients, viral DNA appeared in blood 30 (6–44) days after transplantation, was detected for 116 (30–399) days and reached its peak 44 (18–52) days after transplantation. During follow-up, these patients had negative or very low levels of HCMV-specific CD4^+^ T-cells (median 0.1, range 0.1–0.3 T-cells/µL) for about two-three months after transplantation (see representative case in Fig. 1C), whereas levels of CD8^+^ T-cells were at the cut-off level (median 0.4, range 0.1–11.4), and increased with time, reaching after 120 days, levels significantly higher than those observed at day 30 (from now on, this group will be named CD8^+^ T-cell dominated response). Levels of specific CD4^+^ T-cells in these patients were restored above the cutoff between 90 and 120 days after transplantation, reaching median levels of 0.7 (0.3–2.9) at the end of follow-up, while specific CD8^+^ T-cells reached a median level of 55.9 (21.0–212.6). Viral DNA dropped to a median level of 350 (<25–287,200) copies/mL. Median levels of total CD4^+^ and CD8^+^ T-cells were above 300/µL at the onset of follow-up. Notwithstanding the initial lack of specific CD4^+^ T-cells, also group 3 patients spontaneously controlled HCMV infection in the absence of antiviral therapy. In this respect, it is impressive to look at the kinetics of viral load in a HTR reaching the level of 287,200 copies/mL (just below the cut-off level for antiviral therapy) at 95 days after transplantation concomitantly with onset of HCMV-specific CD4^+^ (and CD8^+^) T-cell reconstitution. Afterwards, viral load regressed until it reached less than 1,000 copies/mL at 150 days after transplantation in the absence of antiviral treatment.

Finally, group 4 included nine patients (3 KTR, and 6 HTR, 3 D^+^R^+^, 2 D^−^R^+^, and 4 R^+^ with D unknown) with a median age of 54 (42–68) years, who developed severe HCMV infection (viral load above the cutoff of 300,000 DNA copies/mL blood) thus requiring pre-emptive therapy (see representative case in Fig. 1D). In these patients, median times to DNA detection onset, duration and peak were 25 (14–37), 182 (50–347), and 48 (41–136) days after transplantation. Three of these patients also suffered from gastrointestinal disease, while two had HCMV-related fever, and four were asymptomatic. These patients had significantly lower total CD4^+^ T-cell levels than patients with self-resolving infection at 60 and 90 days after transplantation (Fig. 2A), and did not show the presence of specific CD4^+^ T-cells until 4–6 months after transplantation (Fig. 2B). However, they had both total and specific CD8^+^ T-cells at the same level as patients with no infection or self-resolving infection (Fig. 3A and B), as well as the nine patients controlling infection with CD8^+^ T-cells alone before the appearance of specific CD4^+^ T-cells. However, as observed also in group 3 patients, specific CD8^+^ T cells increased with time reaching after 120 days, levels significantly higher than those observed at day 30. Antiviral therapy was administered until DNA disappearance from blood, for a median time of 50 (18–136) days and consistently resolved HCMV infection and the relevant symptoms. Concomitantly, at the end of follow-up, the median numbers of specific CD4^+^ T-cells shifted from 0.1 (0.1–0.9) to 0.7 (0.1–6.7), and CD8^+^ T-cells from 0.8 (0.1–8.7) to 29.5 (5.3–323.5).

All patients were alive at the end of follow up, with the exception of two (belonging to groups 3 and 4), who died at 8 and 12 months after transplantation due to non-HCMV related causes.

**Figure 4:**
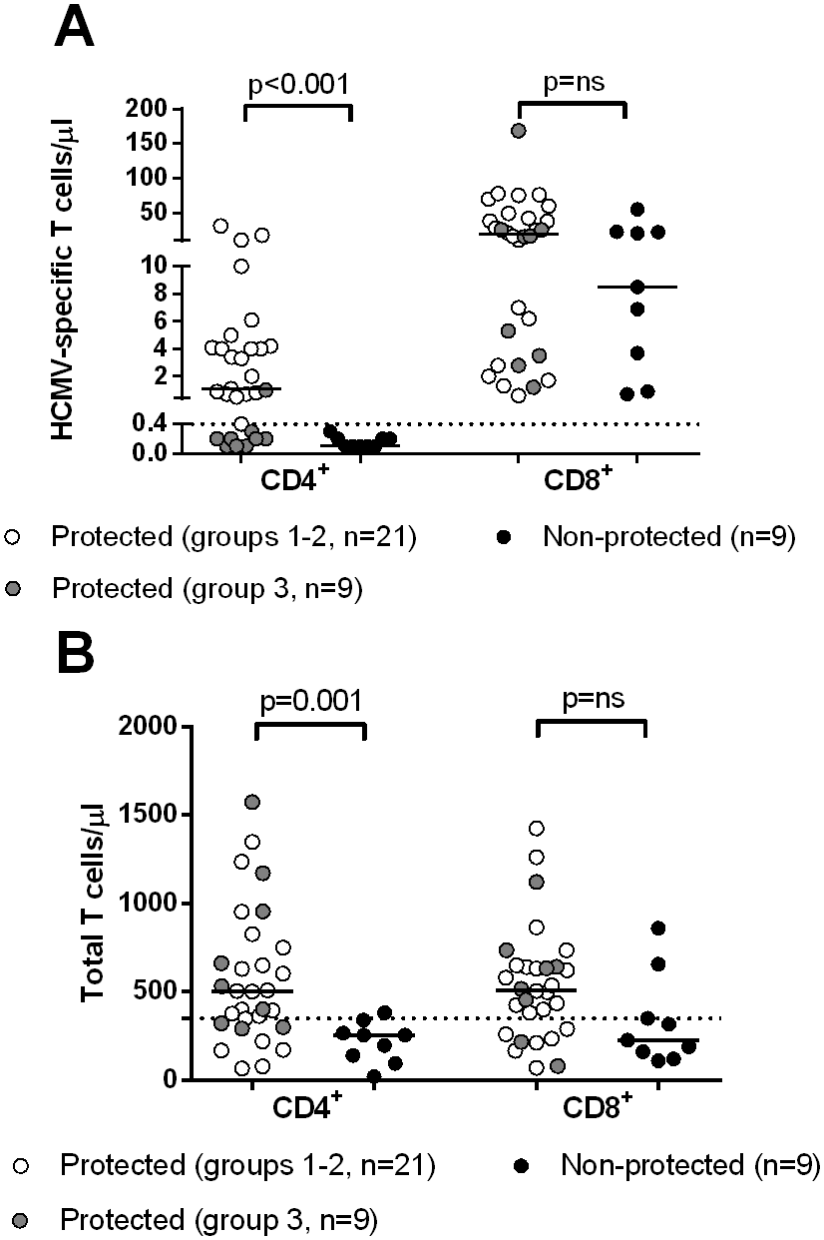
Comparison of (A) HCMV-specific and (B) total T-cells/µl in group 4 non-protected patients ***vs*** **groups 1+2+3 protected patients.** While both total and HCMV-specific CD4^+^ T-cells are significantly higher in protected patients, no difference is observed between protected and non-protected patients for both total and specific CD8^+^ T-cells.

In order to analyze the relationship between control of HCMV infection and total or specific T-cells, we considered the efficacy of the T cell counts determined at 60 days after transplantation (i.e. the time-point closest to the peak of HCMV infection) in discriminating patients with spontaneous control of HCMV infection (groups 1+2+3) from patients with severe infections requiring antiviral treatment (group 4). It was found that both levels of total and HCMV-specific CD4^+^ T cells were significantly higher in patients with spontaneous control of HCMV infection as compared to non-protected patients (Fig. 4A and 4B). In addition, group 4 patients with severe infection were more accurately identified by determination of HCMV-specific rather than total CD4^+^ T-cells (area under the ROC curve at 60 days after transplantation: 0.90 *vs* 0.84). HCMV-specific CD4^+^ T-cells showed a sensitivity of 100% and a specificity of 73% in detecting group 4 patients with severe infections, whereas total CD4^+^ T-cells (with an optimal cut-off of 350 cells/µL) showed a sensitivity of 89% and the same 73% specificity. In more detail, patients with severe HCMV infection had total or specific CD4^+^ T-cell levels significantly lower than patients with self-resolving or no infection. On the contrary, no significant association was found between total or specific CD8^+^ T-cells and control of HCMV infection (Fig. 4 A and B).

### Factors potentially associated with patient distribution into the 4 groups

Within the limits of the small number of patients studied, we analyzed the possible correlation between different factors (age, transplanted organ, induction and immunosuppressive regimens, acute rejection) and the virological and immunological patient distribution into the 4 groups (Table 2). Early specific CD4^+^ and CD8^+^ T cells and negative or low viral load (i.e. groups 1 and 2) were significantly associated with kidney *vs* heart transplantation, and with anti-CD25 *vs* ATG administration. No significant association was found with age, immunosuppressive regimen or development of acute rejection. However, when considering immunosuppressive therapy, most (57%) group 1 patients (with no infection) received RAD (both KTR and HTR), whereas only 20% of all the other patients belonging to groups 2, 3 and 4 (all with HCMV infection) received a RAD containing regimen (p = 0.04).

**Table 2:** Factors potentially associated with patient distribution into the four groups.

Patient groups	Age, median yrs (range)	Transplanted organ (% patients)		Induction therapy (% patients)		Immunosuppressive regimen containing (% patients):		Acute rejection (%)
		Heart (n = 14)	Kidney (n = 25)	ATG^a^ (n = 26)	Anti-CD25 (n = 13)	MMF^b^ (n = 27)	RAD^c^/RAD+MMF low dosage (n = 10)	
1 (n = 7)	50 (43–60)	14	86	57	43	43	57	14
2 (n = 14)	57 (46–71)	14	86	43	57	77	23	7
3 (n = 9)	58 (24–69)	56	44	89	11	75	25	22
4 (n = 9)	54 (42–68)	67	33	89	11	89	11	22
P value	ns^d^	0.02		0.04		ns		ns

a ATG, anti-thymocyte globulin.

b MMF, mophetil mycophenolate.

c RAD, rapamycin derivative.

d ns, not significant.

### Analysis of polyfunctional cytokine production in CD4^+^ and CD8^+^ T-cells

**Figure 5:**
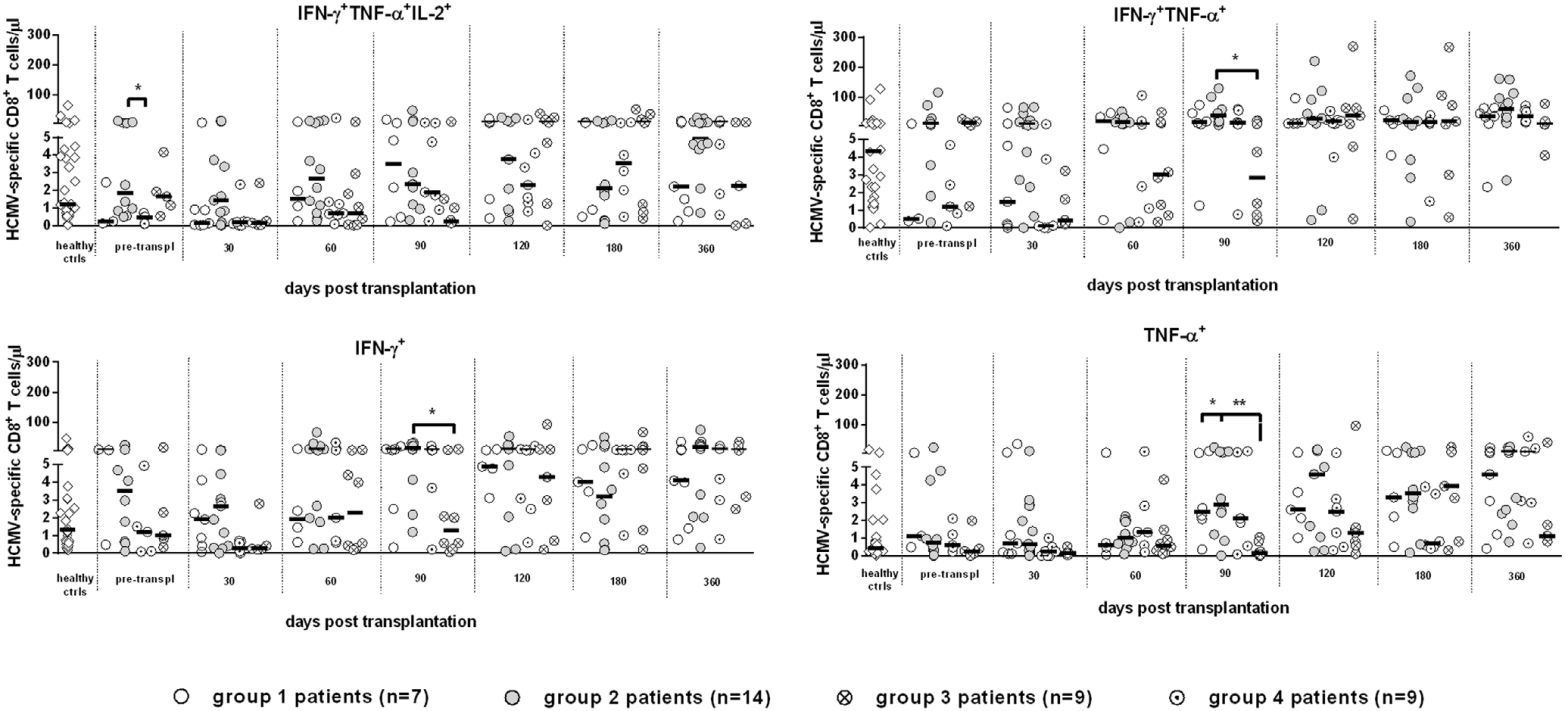
Kinetics of median levels of polyfunctional HCMV-specific CD8^+^ T-cells in groups 1–4 SOTR. No consistent statistically significant difference was observed between the four patient groups.

With the aim of identifying differential functional signals of CD8^+^ T-cells temporarily controlling HCMV infection in group 3 patients in comparison with CD8^+^ T-cells not controlling HCMV infection in patients with high viral load requiring antiviral treatment (group 4), we performed an analysis of polyfunctional CD8^+^ T-cells in the two groups of SOTR. As shown in Fig. 5, the frequency of multifunctional (i.e. producing IFN-γ/TNF-α/IL-2 or IFN-γ/TNF-α) or monofunctional (i.e. producing only IFN-γ or TNF-α) HCMV-specific CD8^+^ T-cells were not substantially different between the two groups. Thus, the multifunctional activity of CD8^+^ T-cells of patients temporarily controlling HCMV infection did not appear to be superior (within the limits of the cytokines tested), to that of patients not controlling the infection. In addition, we examined also the cytokine profile of specific CD8^+^ T cells of groups 1 and 2 (with and without HCMV reactivation in the presence of specific CD4^+^ T cells), and again no difference was found with respect to the presence of polyfunctional T cells in the two groups (Fig. 5).

**Figure 6:**
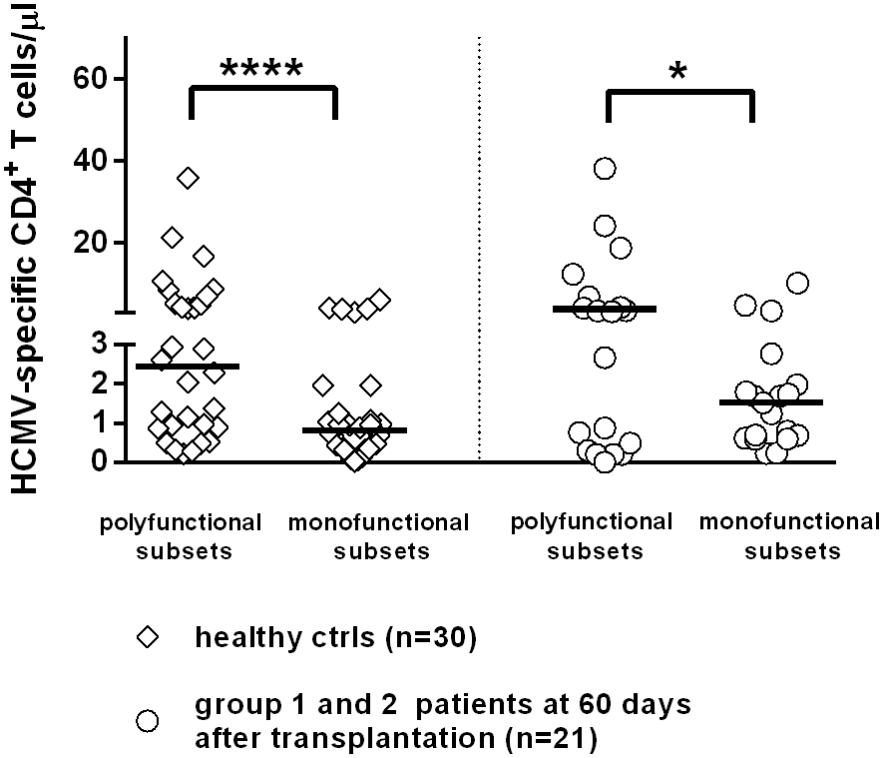
Polyfunctional hierarchy of HCMV-specific CD4^+^ T-cells in groups 1 and 2 SOTR. Predominance of polyfunctional vs monofunctional CD4^+^ T-cells in healthy controls and in protected patients of groups 1+2.

Furthermore, in a subgroup of patients, we also examined perforin expression by HCMV-specific CD8^+^ T-cells: all patients showed high levels (>80%) of HCMV-specific CD8^+^ T-cells expressing perforin, regardless of their ability to control or not HCMV infection (data not shown). Finally, the polyfunctional hierarchy of CD4^+^ T-cells of patient groups 1 and 2 was reported in Fig. 6, showing the predominance of multi-functional CD4^+^ T-cells in these patient groups controlling the infection vs monofunctional CD4^+^ T-cells.

### γδ T-cells and HCMV reactivation in SOTR

**Figure 7:**
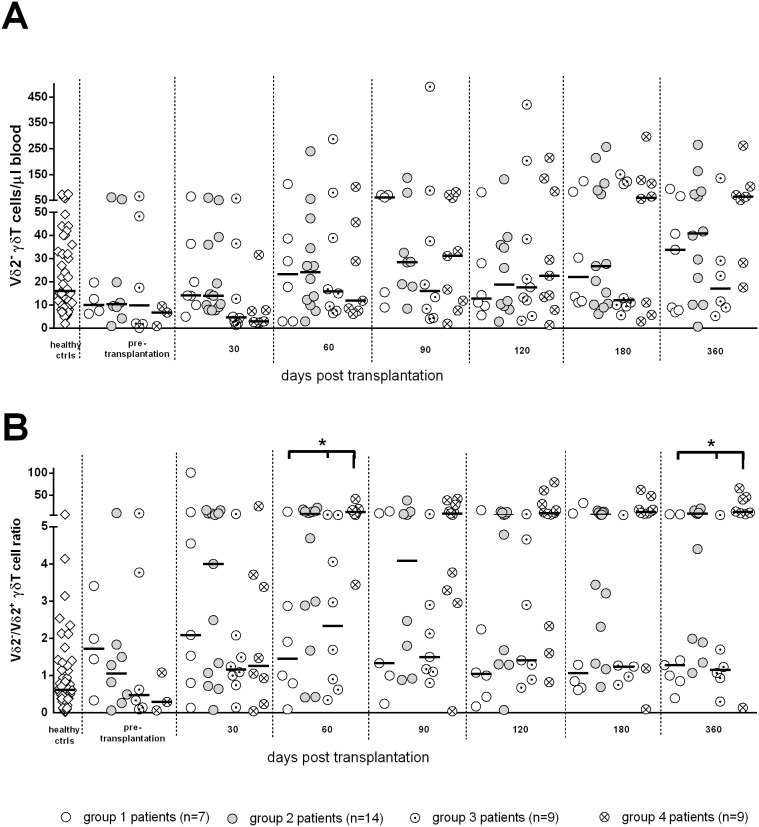
Kinetics of median levels of (A) Vδ2^−^ γδ T-cells and (B) Vδ2^−^/Vδ2^+^ γδ T-cell ratio. No significant difference was observed among groups in the number of Vδ2^−^ γδ T-cells. The Vδ2**^−^**/Vδ2^+^ γδ T-cell ratio was significantly higher in group 4 than in groups 1 and 3 at days 60 and 360. Stars above columns indicate significant differences among transplanted patient groups: *, P<0.05.

When considering the absolute number of Vδ2^−^ γδ T-cells (this subset is reported to expand in response to HCMV infection), no significant difference among groups was observed (Fig. 7A). We then analyzed the Vδ2^−/^Vδ2^+^ ratio in order to evaluate the relative expansion of Vδ2^−^ with respect to Vδ2^+^ γδ T-cells (Fig. 7B). This ratio was significantly higher in group 4 patients than in group 1 and group 3 patients at days 60 and 360, thus indicating a relative expansion of Vδ2^−^ γδ T-cells in response to HCMV infection, particularly in patients with severe infection (group 4).

## Discussion

Results of the present study indicate that in HCMV-seropositive immunosuppressed transplanted patients complete immune reconstitution, in association with protection from HCMV infection reactivation, occurs only when HCMV-specific CD4^+^ T-cells reconstitute their functions and provide help to specific CD8^+^ T-cells. The relatively short time lapse from when HCMV-specific CD8^+^ T-cells appear to control HCMV infection alone (group 3 patients) is consistently followed by reconstitution of HCMV-specific CD4^+^ T-cells.

In this study, we examined, based on viral load, 39 SOTR. Four groups of patients were identified. Group 1 included patients with no HCMV reactivation in the absence of viral DNA in blood. Group 2 patients were affected by a controlled HCMV reactivation with low-level viral load in blood. Group 3 included patients controlling HCMV infection with moderate levels of viral DNA and absence of specific CD4^+^ T cells at the beginning of follow-up, while group 4 included patients suffering from severe HCMV infection with very high viral load and, thus, requiring antiviral treatment.

Group 3 patients controlled HCMV reactivation for the first three months after transplantation in the presence of HCMV-specific CD8^+^ T-cells only, while specific CD4^+^ T-cells were not detected or detected at a level below the cutoff (CD8^+^ dominated T-cell response). Viral load reached variable levels below the viral cutoff, but the infection appeared to be controlled only after the first three months when specific CD4^+^ T-cells appeared and a drop in viral load was observed. In group 3 patients controlling the infection in the presence of CD8^+^ T-cells only, it is possible that low levels of CD4^+^ were already present in the blood or other CD4^+^ T-cells residing in lymphoid organs may have played a role in protection in the first period after transplantation.

Group 4 patients controlled the infection thanks to antiviral treatment, which allowed the CD4^+^ T-cell response to reconstitute prior to worsening of disease. Specific CD8^+^ T-cells were not sufficient to control the infection from its onset. Peak viral load was reached at about the same time after transplantation as in group 3 patients. However, viral load was much higher reaching the cutoff for antiviral treatment; this appears to be due to the more delayed appearance of specific CD4^+^ T-cells. In some patients from this group, clinicians started antiviral therapy prior to reaching the viral DNA cut-off of 300,000 copies/mL blood. In these patients, early antiviral intervention was required due to the concomitant presence of HCMV gastrointestinal disease, which caused a dissociation between viral load in blood and local viral load. In this respect, it has recently been shown that HCMV end-organ disease may be associated with high or low viral load in blood in pre-emptively treated SOTR [20].

It should be noted that, although not significant, there was a trend towards HCMV-specific CD8^+^ increase in all four groups, including patients with low or no HCMV in blood. This suggests that also in patients without detectable HCMV in blood, low level HCMV reactivation may occur and stimulate specific CD8^+^ expansion.

As for patients’ characteristics, it was found that KTR and patients receiving anti-CD25 had a better CD4^+^ T cell response and control of HCMV infection than HTR or KTR patients receiving ATG. In addition, RAD-treated patients showed a trend towards a lower incidence of HCMV infection. These results are in line with previous studies, which report a reduction in the rate of HCMV infection, as well as an earlier T-cell response in RAD-treated KTR and HTR patients [19], [24], [25].

CD4^+^ T-cells have several roles in the cell-mediated immune response and do not seem to be replaced by other functions/cells in the post-transplant period. The CD4^+^ T-cell helper function is directed to B-cells and CD8^+^ T-cells. Initially, in the mouse model following CD8^+^ T-cell depletion prior to animal infection, CD4^+^ T-cells were found to be critically involved in the control of virus replication [26]. Subsequently, the role of CD4^+^ T-cells was shown to be critical both in the immunocompetent and the immunocompromised host. In the immunocompetent (healthy children), it was found that an impaired CD4^+^ T-cell response was associated with prolonged periods of virus shedding both in urine and saliva [10]. In addition, in primary infections of KTR, it was shown that asymptomatic infections were associated with appearance of IFN-γ-producing CD4^+^ T-cells, prior to the emergence of CD8^+^ T-cells [9]. Conversely, in the same study it was found that a delay in the appearance of HCMV-specific CD4^+^ T-cells was accompanied by extended virus replication and viral disease. In other studies, it was found that decreased levels of HCMV-specific CD4^+^ T-cells were detected prior to an increase in viral load and onset of clinical disease both in renal transplant as well as lung transplant recipients [11], [27]. Furthermore, adoptive T-cell immunotherapy interventions showed that maintenance of HCMV-specific CD8^+^ T-cells infused after bone marrow transplantation required the presence of HCMV-specific CD4^+^ T-cells, further suggesting a critical role for CD4^+^ T-cells during HCMV infection [6], [8]. Thus, it seems that CD4^+^ T cell help is mandatory for CD8^+^ T cells to exploit their optimal effector function against persistent viral infections, which can be resolved only in the presence of both specific CD4^+^ and CD8^+^ T cells, as observed in the mouse model [28]–[30].

Our conclusion is that functional activity of HCMV-specific CD8^+^ T-cells may be fully exerted only if assisted by HCMV-specific CD4^+^ T-cells, not only in the induction, but also in the maintenance phase.

In the last decade, it has been reported that IE-1-specific T-cells confer protection against HCMV reactivation, whereas pp65-specific T-cells do not prevent HCMV disease [31], [32]. In our Center, both the development and reconstitution of the T-cell response to IE-1 and pp65 peptide mixtures have been observed to underestimate the level of the T-cell responses [33], [34]. In other studies, no correlation between IE-1 and pp65-specific T-cells and protection from HCMV disease has been reported [35]. In the meantime, a large pool of predefined epitopic peptides from multiple HCMV proteins was proposed for prompt stimulation of CD8^+^ T-cells, thus reducing the underestimation of the actual level of immune protection against HCMV with respect to assays using IE-1 and pp65 peptide mixtures alone [36].

It is currently believed that polyfunctional T-cell responses may be used as markers of effective antiviral immunity [37]. In particular, multiple cytokine-producing antiviral CD4^+^ T-cells have been shown to be functionally superior to single cytokine-producing cells, thus suggesting that vaccines should aim to elicit T-cells that produce more than one cytokine [38]. However, in our study, when we compared the polyfunctional activity of CD8^+^ T-cells in SOTR temporarily controlling the infection in the presence of CD8^+^ T-cells only (group 3 patients) *vs* patients developing severe HCMV infection (and even disease) and requiring antiviral treatment (group 4 patients), we did not observe any significant difference in the frequency of multi- *vs* mono-functional CD8^+^ T-cells between the two patient groups.

As for γδ T-cells, these cells have been reported to represent a first-line defense mechanism against HCMV infection in kidney allograft recipients [12]. Expansion of γδ T-cells was observed concomitantly with the resolution of HCMV infection and disease, regardless of the HCMV serologic status of the donor and recipient prior to transplantation [13]. Only Vδ2^−^ γδ T-cells underwent long-lasting expansion and were able to kill HCMV-infected target cells in vitro [14]. In addition, long-term expansion of effector/memory Vδ2^−^ γδ T-cells has been shown to be a specific signature of an adaptive immune response to HCMV infection in both immunocompetent and immunocompromised patients [39], [40]. In the present study, we can confirm at least a relative expansion of Vδ2^−^ with respect of Vδ2^+^ γδ T-cells in patients with HCMV reactivation after SOT. It is difficult to attribute a protective role to this subset, since the expansion was even more pronounced in patients with severe HCMV infection than in patients with self-resolving infection. Our observations suggest more a “reactive” than a “protective” role for Vδ2^−^ γδ T-cell expansion after HCMV reactivation in HCMV-seropositive SOTR.

In this study, we adopted a method enabling the simultaneous determination of both HCMV-specific CD4^+^ and CD8^+^ T cell responses [20], but it seems that measurement of HCMV-specific CD4^+^ T-cells alone might be sufficient to define protection from HCMV infection. Thus, simpler methods for the *ex vivo* detection of HCMV-specific CD4^+^ T-cells could be adopted in clinical practice [41]. In this respect, the measurement of the total CD4^+^ T-cell count could provide a general indication on the patient’s risk to develop an infectious complication after transplantation [42]. However, several patients with or without severe HCMV infections showed overlapping levels of total CD4^+^ T-cells, whereas determination of HCMV-specific CD4^+^ T-cells provided more targeted and reliable information for the management of HCMV infection complications. Conversely, the clinical reliability of methods, such as ELISpot [43] or QuantiFERON [44] determining HCMV-specific CD8^+^ T-cells only, appears questionable in view of these data for precise clinical immunologic monitoring of HCMV infection in transplant recipients.

In conclusion, results of our study consistently document that in two of four transplanted patient populations examined, long-term protection from HCMV infection was reached only in the presence of HCMV-specific CD4^+^ T-cells above the established cutoff (and always in association with HCMV-specific CD8^+^ T-cells), as we preliminarily observed (19). On the other hand, CD8^+^ T-cells alone did not confer long-lasting immune control of HCMV infection in all patients examined. However, other potential factors enabling the short-term control of HCMV by CD8^+^ T-cells should be further investigated, such as roles of innate and humoral immunity.
